# Polymer-derived distance penalties improve chromatin interaction predictions from single-cell data across crop genomes

**DOI:** 10.1101/2025.08.20.671329

**Published:** 2025-08-23

**Authors:** Luca Schlegel, Fabio Gómez Cano, Alexandre P. Marand, Frank Johannes

**Affiliations:** 1 Plant Epigenomics, TUM School of Life Sciences Weihenstephan, Technical University of Munich, Freising, 85354, Germany; 2 Department of Molecular, Cellular, and Developmental Biology, University of Michigan, Ann Arbor, MI 48109, USA

## Abstract

Scalable proxies of 3D genome interactions, such as from single-cell co-accessibility or Deep Learning, systematically overestimate long-range chromatin contacts. To correct this bias, we introduce a penalty function grounded in polymer physics, derived by fitting a multi-component power-law model to experimental Hi-C data from maize, rice, and soybean. This correction substantially improves concordance with Hi-C, reduces false-positive rates of long-range interactions by up to 95%, and reveals distinct decay exponents corresponding to different scales of chromatin organization. We provide open-source code and derived parameters to facilitate broad application across plant species.

## Introduction

The three-dimensional (3D) organization of chromatin is essential for gene regulation and genome stability in eukaryotes [1–3]. At the finest resolution, chromatin loops connect distal genomic loci, such as enhancers and promoters, to close spatial proximity to control gene expression [4–6]. In many plant species, these architectural features are nested within larger Topologically Associating Domains (TADs) [7–9], which are themselves organized into active (A) and inactive (B) compartments [1, 10–13]. While genome-wide methods like Hi-C have been instrumental in mapping this hierarchy [10], their application in large-scale comparative studies is limited by their resource-intense nature [14].

Consequently, scalable proxies such as co-accessibility scores from single-cell ATAC-seq (scATAC-seq) [15, 16] and predictions from deep learning (DL) models [17, 18] have emerged as alternative approaches for charting chromatin interactions across diverse cell types and conditions. Co-accessibility infers contacts by identifying pairs of genomic loci whose accessibility patterns covary across individual cells, while DL approaches learn sequence-based features predictive of physical proximity from experimental training data. These methods promise high throughput and resolution without the expense or logistical hurdles of genome-wide 3D mapping. However, both approaches systematically overestimate long-range contacts [19], thus introducing biases in the 3D representation of the genome. Because co-accessibility relies on pairwise correlation of accessibility profiles, loci co-regulated by the same transcription factors, chromatin remodelers, or small RNA pathways [20] can appear "connected" even when they are spatially distant, inflating false positives at large genomic separations. Similarly, DL models trained on Hi-C or Hi-ChIP data learn sequence motifs and epigenomic signals that correlate with interaction frequency but do not explicitly incorporate genomic distance as a feature [17, 19, 21]. As a result, these models tend to predict spurious distal contacts, contradicting the well-established power-law decay of contact probability with increasing genomic distance observed in both Hi-C experiments and polymer physics theory [10, 22–24]. Although heuristic normalization schemes have been introduced to partially correct for this bias [25], a principled, physics-based penalty function remains lacking.

To develop such a principled penalty function, we turn to the field of polymer physics modeling. This field offers two main computational approaches, and can be broadly classified as ‘forward’ (mechanistic), which test assumed physical rules like epigenomic-driven interactions of loop extrusions [26–29], or ‘inverse’ (data-driven), which infer 3D structures directly from experimental data [30–35]. Hybrid approaches also exist that combine these philosophies, for example, by using iterative algorithms to learn the strength of mechanistic forces driving genome organization, such as the attraction between chromatin compartments, by comparing simulated contact maps directly to experimental Hi-C data [36]. While these approaches are increasingly common [37], their application to plant genomes remains less explored [38, 39], particularly in species with large, complex genomes that exhibit unique architectural features [40, 41].

To address this gap, we developed a data-driven framework that applies species-specific, polymer-derived penalty functions to existing co-accessibility and DL-based interaction scores. By fitting a multi-component power-law model to Hi-C-derived contact decay profiles in rice, soybean, and maize, we extract distinct scaling exponents that capture the heterogeneous folding regimes of each genome. Incorporating these exponents into a distance-dependent penalty not only restores the expected decay behavior but also reduces long-range false positives by up to 95% on average, bringing both scATAC-seq co-accessibility and DL predictions into concordance with experimental data. This approach offers an easy-to-implement correction grounded in first principles and provides effective default parameters for application to other species.

## Results and Discussion

### Polymer-based Power-law decay of Hi-C contacts reveals a multi-scale architecture in plants

**Figure 1:**
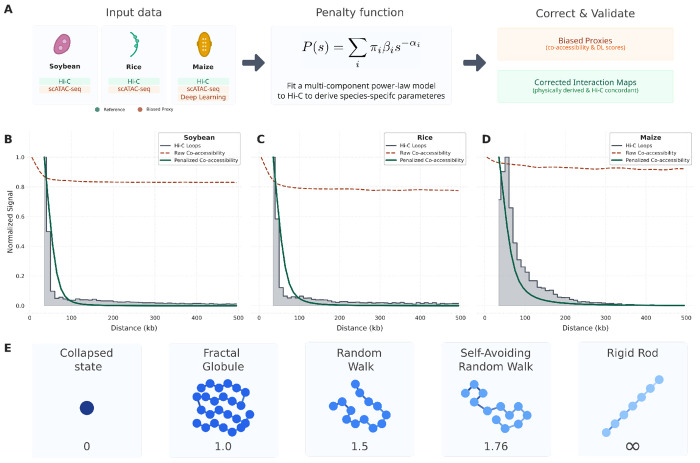
A polymer physics-based penalty corrects distance bias in co-accessibility scores. **(A)** Conceptual workflow: A multi-component power-law model is fitted to species-specific Hi-C data to derive a penalty function. This function corrects biased proxies by down-weighting long-range interactions. **(B-D)** Chromatin interaction profiles for soybean (B), rice (C), and maize (D). In each species, the Hi-C loop decay profile (grey histogram) shows the expected decrease in interaction frequency with genomic distance. In contrast, raw co-accessibility scores between Accessible Chromatin Regions (ACRs) are largely distance-independent (dark red dashed line), overestimating long-range contacts. Applying our penalty function corrects this bias, producing a penalized co-accessibility profile (dark green solid line) that closely matches the Hi-C reference. All profiles are normalized to a maximum of 1.0. Hi-C loop data is restricted to interactions >35 kb. **(E)** The power-law decay exponent *α* corresponds to distinct theoretical polymer configurations. These idealized models, from a collapsed state (*α* = 0) to a rigid rod (*α* = ∞), provide a framework for interpreting the different decay exponents and folding regimes observed in empirical data.

The interaction frequency between genomic loci is fundamentally constrained by the physical properties of chromatin, which exhibit polymer-like behavior. Theoretical models of polymer physics predict that the contact probability *P*(*s*) between two points on a polymer chain decays as a power-law function of their linear distance *s* following the relationship *P*(*s*) ≈ *s^−α^*, with *α* > 0 (see Methods). While this geometric framework establishes the power-law relationship, it does not provide a universal value for *α*, which is known to be dependent on species and genomic context [42, 43]. The value of *α* can be derived from theoretical first principles or determined empirically from experimental data. Polymer simulations show that different values of *α* correspond to distinct folding regimes, ranging from a fully collapsed state (*α* = 0) and the Fractal Globule (*α* ≈ 1.0) to a simple random walk in 3D (*α* = 1.5) (Figure 1E). The Fractal Globule model is particularly relevant as it describes a dense, unentangled, “knot-free“ state that allows chromatin to be tightly packed while keeping individual loci accessible for DNA-templated processes, reproducing key properties of eukaryotic chromosome organization [4]. However, empirical measurements reveal a more complex picture. For instance, early reports in humans reported a Fractal Globule configuration (*α* ≈ 1.08), but subsequent analyses identified distinct exponents for intra-domain scaling (*α* ≈ 0.76) and an updated genome-wide average of *α* ≈ 1.27 [44]. In plants like sugarcane and potato, power law decays of the form *P*(*s*) = *β* · *s^−α^* have also been observed with decay exponents *α* between 0.8 and 0.9, but this analysis was performed on individual, unscaffolded contigs in the context of validating data for genome scaffolding [45]. Notably, it was observed that the scaling factor *β* in this relationship varied considerably with sequencing depth and the amount of data collected.

**Figure 2:**
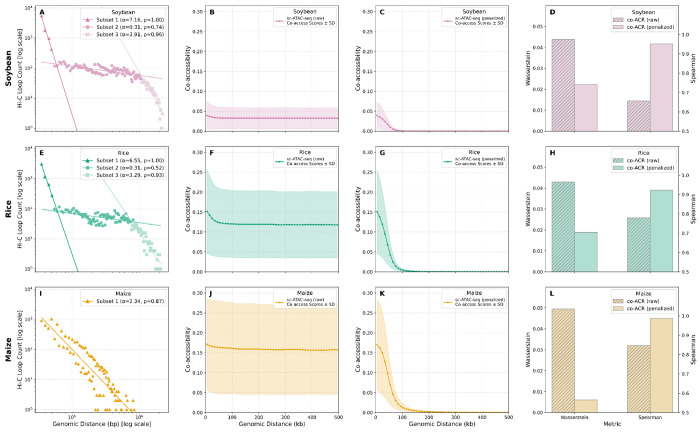
Derivation and statistical validation of species-specific penalty functions. **(A, E, I)** Species-specific penalty functions are derived by decomposing log-log plots of Hi-C loop counts using a Gaussian Mixture Model (GMM). This method identifies distinct subsets of interactions (distinguished by color and marker style), each with a unique power-law decay exponent (*α*) that informs the penalty. **(B, F, J)** Raw co-accessibility scores show little to no decay with increasing genomic distance. **(C, G, K)** Applying the species-specific penalty transforms the raw scores, producing penalized profiles that exhibit a sharp, Hi-C-like decay. In (B, F, J) and (C, G, K), the shaded areas represent the standard deviation of scores across all ACR pairs. **(D, H, L)** The correction is validated statistically for soybean (D), rice (H), and maize (L). Penalized scores (solid bars) show a substantially improved concordance with Hi-C data compared to raw scores (hatched bars), confirmed by a lower Wasserstein distance and a higher Spearman correlation.

Given this complexity, we opted for a direct empirical approach to determine the decay characteristics in our target species. To do this, we re-analyzed Hi-C data in soybean [46], rice [47], and maize [48] leaf samples using a uniform pipeline of HiC-Pro and HICCUPs for raw read alignment, processing, normalization, and chromatin loop calling with fixed parameters. Plotting the resulting Hi-C contact frequency versus genomic distance on a log-log scale, revealed that the scaling is only piecewise linear. This observation suggests that a single decay exponent cannot capture the full complexity of empirical chromatin contact data, which is consistent with previous experimental and theoretical studies [29, 44]. To account for this heterogeneity, we employed a Gaussian Mixture Model (GMM) to decompose the interaction profiles into distinct subpopulations (Figure 2A, E, I). This approach enabled the fitting of a unique set of decay exponents {*α_i_*} for each species, revealing three distinct components for rice and soybean but only a single component for maize (see Methods and Supplementary Table S1).

**Figure 3:**
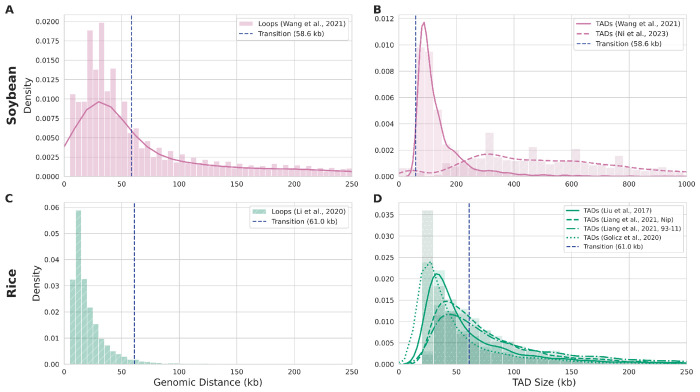
GMM-derived transition points delineate characteristic scales of loops and TADs in soybean and rice. **(A, B)** Soybean: The 58.6 kb threshold coincides with the steepest decay in loop counts, marking the shift from abundant short-range contacts to fewer medium- and long-range interactions (KDE minimum at ~56.4 kb; n = 32,181). For TADs, the transition point defines the lower boundary of domain size, with ≥98% of TADs larger than 58.6 kb (Wang 2021: 100%, Ni 2023: 96.4%). **(C, D)** Rice: The 61.0 kb threshold acts as an upper limit, with ~94% of loops at or below this distance (n = 7,726). For TADs, a the transition point approximates the median size across independent studies (median sizes ranging from 35 kb to 70 kb [47, 53, 54]), placing 61.0 kb near the central tendency of these values.

In rice and soybean, the first folding regime, spanning genomic distances from 35kb to ~60kb, was characterized by a steep, short-range exponent (*α* = 6.6 and *α* = 7.2, respectively). Such high exponents are geometrically consistent with chromatin being organized into a series of tightly packed, partitioned globules that strongly suppress long-range contacts (Figure 1E). In both soybean and rice, the GMM-derived transition point coincides with the characteristic scale of loops; for rice, the 61.0 kb threshold acts as a clear upper bound, containing >94% of all identified loops [49], while for soybean [46], the 58.6 kb threshold aligns with the steepest decay of the loop distribution (Figure 3A,C). This is analogous to general principles of eukaryotic genome organization, such as the Multi-Loop-Subcompartment (MLS) architecture in mammals, where ~100 kb loop rosettes form dense hubs of regulatory interactions [50], and is consistent with known sizes of functional loops and sub-TADs in plants [51].

Transitioning to the second regime (~60 kb to ~1 Mb), the decay exponent becomes remarkably shallow for both species (*α* = 0.31). This is a signature of large-scale organization into Topologically Associating Domains (TADs) with embedded A/B compartments [49]. In soybean, our analysis reveals that the 58.6 kb transition point acts as a sharp lower boundary for TADs. Across two independent studies [46, 52], we found that over 98% of all identified TADs were larger than this threshold, separating them from the smaller loop-based domain (Figure 3B). In rice, the 61.0 kb transition point does not act as a sharp boundary but instead approximates the median TAD size across multiple independent studies [47, 53, 54], which show median sizes ranging from 35 kb to 70 kb (Figure 3D). A similar observation was reported by Kurbidaeva et al., who measured a median TAD size of 63 kb at 5 kb resolution using an independent Micro-C protocol [55]. For both species, the shallow exponent suggests the formation of a collapsed, ”well-mixed” polymer globule (Figure 1E). This state is likely driven by a combination of physical mechanisms, ranging from processes like phase separation and depletion attraction [39], to more specific interactions, such as the self-attraction of facultative heterochromatin shown to drive local compaction in Arabidopsis simulations [56]. This interpretation is further strengthened by dynamic polymer models, which confirm that low scaling exponents approaching zero are characteristic of such dense, collapsed heterochromatin states [29].

In contrast to rice and soybean, the maize genome was best described by a single power-law decay with *α* = 2.34. This value is notably similar to the exponent of *α* = 2.1 reported for open randomly folded euchromatin in ”strings and binders” simulations [29], suggesting a more fluid chromatin architecture with less sharply defined domain boundaries, a hypothesis consistent with the highly complex and repeat-dense nature of the maize genome [57–59]. This model of a fluid architecture is consistent with recent findings using Hi-C and 3D-FISH microscopy, which showed that maize euchromatin is a balanced, fine-grained mixture of early- and middle-replicating subcompartments with opposing properties of chromatin condensation and interaction range [60].

### Co-accessibility scores and Deep Learning predictions overestimate long-range interactions

To explore to which extent single cell co-accessibility maps could recapitulate the 3D interaction distances from the Hi-C data, we re-analyzed available single-cell ATAC-seq (scATAC-seq) data from soybean [61], rice [62], and maize [25]. Following standard preprocessing of scATAC-seq data sets, we generated pseudocells using a data-driven k-nearest neighbors approach to overcome sparsity and statistical limitations of binary data [25]. Next, we leveraged pairwise Spearman correlation coefficients of normalized accessibility values among Accessible Chromatin Regions (ACRs) within 2 and 500 kb across pseudocells, revealing a total of 6.3 million co-accessible ACRs in soybean, 3.8 million in rice, and 3.4 million in maize with positive co-accessibility values, suggestive of chromatin interactions. A side-by-side comparison with the Hi-C data revealed systematic biases in these interaction proxies: the signal fails to decay with increasing genomic distance (Figure 1B–D, dark red dashed lines). This lack of distance dependency leads to a significant overestimation of long-range interactions compared to the power-law decay observed in Hi-C data. We quantified this bias using the Hi-C profile as the experimental ground truth. The raw co-accessibility profiles showed low concordance with Hi-C, exhibiting low Spearman correlations and high Wasserstein distances (Figure 2D, H, L). To measure the signal disagreement, we calculated an area-based False Positive Rate (FPR) and False Negative Rate (FNR) by comparing the proxy and reference curves (see Methods and Supplementary Table S2). The raw co-accessibility scores exhibited high FPR of 87.2% (maize), 93.6% (rice), and 94.5% (soybean), highlighting the need for bias correction.

**Figure 4:**
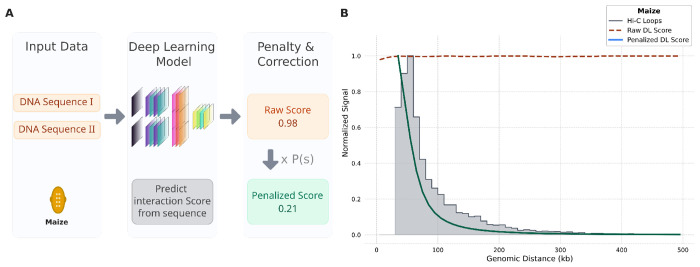
A polymer physics-based penalty corrects distance bias in Deep Learning predictions for maize. **(A)** Workflow for correcting Deep Learning (DL) scores. The GenomicLinks model processes DNA sequences from a pair of loop anchors to predict a raw interaction score. This score is then corrected using the species-specific penalty function derived from Hi-C data. **(B)** The penalty function effectively corrects DL predictions in maize. The Hi-C loop decay profile (grey histogram) serves as the reference. Raw DL scores (dark red dashed line) are largely independent of genomic distance, overestimating long-range contacts. Applying the polymer-based penalty produces a corrected profile (dark green solid line) that closely matches the Hi-C data. All profiles are normalized to a maximum of 1.0.

In addition to single cell accessibility approaches, Deep learning (DL) has emerged as a powerful approach for predicting chromatin interactions from DNA sequence features, complementing experimental and accessibility-based methods. These models are typically built on architectures combining Convolutional Neural Networks and Long Short-Term Memory networks (CNNs + LSTMs) [17, 21]. By analyzing pairs of genomic loci, they learn to recognize predictive sequence motifs, such as those for insulator proteins like CTCF, to forecast interaction frequencies. However, these common DL architectures do not explicitly incorporate genomic distance as a feature during the training process [19, 63–67]. This forces the model to learn specific sequence-based motifs predictive of interactions but remain agnostic to spatial constraints, resulting in a systematic over-prediction of long-range contacts and a high False Positive Rate (FPR). This phenomenon is not species-specific, but can be observed in models developed for humans [68] as well as in plants [19] (Figure 4A, Supplementary Figure S3). To illustrate this problem, we used the output of GenomicLinks, a DL model that predicts chromatin interactions from DNA sequence for maize [19]. We observed that the raw prediction scores from this model were largely independent of genomic distance, exhibiting a systematic bias similar to that seen in scATAC-seq co-accessibility data (Figure 4A, dark red dashed line).

### A polymer physics-based penalty corrects distance bias in co-accessibility and DL predictions

Previous approaches have sought to correct for the systematic overestimation of long-range contacts. Tools like ArchR [69] and Signac [70], compare the correlation of a given pair of sites against a null distribution generated from background sites matched for genomic distance, overall accessibility, and GC content. A different strategy is used by Cicero [15], which heuristically down-weights long-range interactions in human scATAC-seq co-accessibility scores. Its penalty function is based on the assumption of a single, universal distance-decay exponent *α* = 0.75 with the formula *β* · (1 − *s^−α^*). The corresponding scaling factor *β* is then manually “tuned” to be just strong enough to penalize the vast majority (>95%) of interactions beyond a user-defined distance (default: 250 kb). A direct application of this method to plant genomes is problematic as the exponent is derived from intra-domain contacts in human cells and the heuristic distance cutoff does not necessarily match the distance ranges found in plants.

To overcome these limitations, we derived a penalty function from our GMM-fitted decay exponents {*α_i_*} specific to each plant species (see Methods). This function selectively down-weights long-range interactions, effectively reshaping the co-accessibility profile. We found that applying this function results in a penalized co-accessibility curve that closely mirrors the experimental Hi-C reference profile across all three species (Figure 1B–D, dark green solid line). The penalized profiles show a strong monotonic relationship with Hi-C data, with Spearman correlations *ρ* increasing to an average of 0.95. Similarly, the Wasserstein distance decreased by an average factor of 4.5, indicating a much higher similarity in the overall shape of the decay curves (Figure 2D, H, L). Applying our penalty function substantially improved specificity, reducing the FPR to 4.5% (maize), 32.4% (rice), and to 35.1% (soybean). This corresponds to a 3- to 19-fold reduction in false-positive signals across the three species. Our penalty function reshapes score distributions to reduce the number of pairs passing a given significance threshold (Supplementary Figure S3). While this was accompanied by an increase in the False Negative Rate (FNR), the overall performance, as measured by the F1 score, demonstrates a clear net gain. The average F1 score increased from 0.15 for the raw data to 0.68 after penalization, confirming the value of these penalized co-accessibility scores.

**Figure 5:**
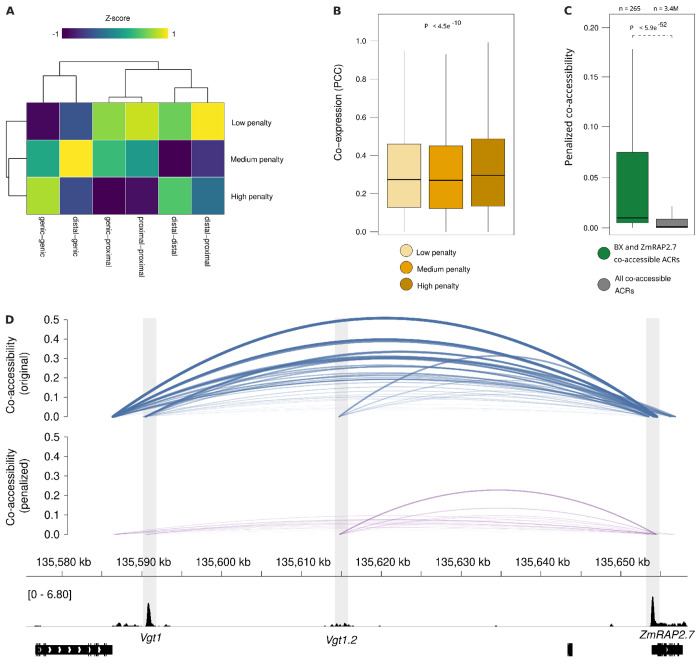
The polymer-physics penalty enriches for functional interactions and distinguishes co-regulation from physical proximity in maize. **(A)** Heatmap of co-accessible ACR classification enrichment (Z-score) in low, medium, and high penalized tertiles. Proximal-distal links are enriched in the low penalty group, while genic-genic links are most penalized. **(B)** Boxplots showing that gene pairs with the highest penalty scores also have the highest co-expression (PCC, Pearson Correlation Coefficient), suggesting the penalty effectively down-weights co-regulated, non-interacting loci. **(C)** Known functional long-range interactions (at the *BX1* and *ZmRAP2.7* loci) retain significantly higher penalized co-accessibility scores compared to the global set of all co-accessible ACRs. **(D)** Genome browser view of raw (top) and penalized (middle) co-accessibility scores, and chromatin accessibility profiles (bottom) at the *Vgt1-ZmRAP2.7* locus. Vgt1, Vegetative to generative transition 1. ZmRAP2.7, Zea mays RELATED TO APETALA2.

We posited that our polymer physics distance penalty down-weights non-functional interactions. To test this, we split co-accessible ACRs into three equal-sized groups – low, medium, and high – based on the difference between the original and penalized co-accessibility scores (e.g. delta co-accessibility scores) using the maize leaf scATAC-seq data set. Classifying co-accessible ACRs by the genomic context of ACR anchors indicated an enrichment of genic-genic and distal-distal links in the most penalized tertile of co-accessible ACRs, while distal-proximal links were associated with the lowest penalized tertile (Figure 5A). Next, we hypothesized that highly penalized co-accessible ACRs reflect co-regulated genes rather than chromatin interactions. Comparing distributions of co-expression values for cognate genic-genic co-accessible ACRs revealed significantly higher co-expression values for the most penalized tertile (Kruskal Wallis rank sum test, P < 4.5e-10; Figure 5B). Down-weighting of co-regulated linkages suggests that our penalty function may be enriched for true interactions. To this end, we evaluated known genetically mapped long-range cis-regulatory interactions associated with *ZmRAP2.7* [71] and *benzoxazin1* (*bx1*) [72]. We found that co-accessible ACRs mapping to *ZmRAP2.7* and *bx1* retain high levels of co-accessibility following distance-based penalization (Wilcoxon rank sum test, P < 5.9e-52; Figure 5C). For example, *Vgt1* and *Vgt1.2* [73] regulatory loci exhibit penalized co-accessibility signal above background with *ZmRAP2.7* (Figure 5D). However, comparing penalized co-accessibility at this locus shows that *Vgt1* is down-weighted compared to *Vgt1.2*, despite *Vgt1* being the primary functional element of *ZmRAP2.7* expression levels (Figure 5D). Overall, these results suggest that our distance-based penalty enriches for functional interactions by depleting co-regulated linkages, but may be conservative for extraordinarily long-range interactions.

To demonstrate the broader utility of our penalty framework, we applied the same species-specific penalty function derived from maize Hi-C data to the GenomicLinks model. Applying the penalty successfully corrected this bias, and the resulting penalized DL scores closely recapitulated the characteristic Hi-C decay profile (Figure 4A, dark green solid line). This successful application to a sequence-based prediction model confirms that our physics-based penalty is a modular and broadly applicable tool for correcting distance bias in diverse proxies of chromatin interaction.

## Conclusion

We have introduced a modular framework, grounded in polymer physics, that corrects the systematic distance bias in proxy measurements of chromatin architecture. The primary strength of this method is its broad applicability as a simple, transparent post-processing step for diverse data types, including co-accessibility scores and deep learning predictions. To facilitate its adoption, we provide our empirically derived parameter sets and open-source code, offering the community a practical tool to enhance the physical realism of chromatin interaction maps [github.com/polymer-penalty].

While our framework provides a robust, static correction based on equilibrium polymer physics, it has limitations. The penalty is purely distance-dependent and does not explicitly model other factors like DNA sequence motifs, protein-mediated looping, or stochastic effects. Furthermore, its reliance on bulk Hi-C data for calibration presents opportunities for future refinement. The current penalty reflects the average state across many cells and may not capture architectural variations between distinct cell types. Key future directions include integrating our static parameters into emerging active polymer models [74], deriving cell-type-specific penalties as single-cell 3D genomics data become more prevalent, and applying our correction to deep learning outputs to create more physically-aware predictive tools.

## Methods

### Data sources

All Hi-C and single-cell ATAC sequencing (scATAC-seq) datasets used in this study were publicly available and re-analyzed for consistent comparison. We obtained data from leaf tissue for soybean (*Glycine max*), rice (*Oryza sativa*), and maize (*Zea mays*). A full list of accession numbers is provided in Supplementary Table S3.

### Hi-C data processing and loop calling:

Raw Hi-C data [46–48]were processed using a uniform HiC-Pro (v3.1.0) [75] pipeline for raw read alignment, normalization, and matrix generation with non-default settings. Specifically, reads were aligned with Bowtie2 in two passes - global followed by local - using --very-sensitive --score-min L, −0.6, −0.2, with seed length −L 30 (global) and −L 20 (local). Alignments with MAPQ < 5 were discarded. A DpnII digestion model was used (ligation motif GATCGATC) with the corresponding fragment BED; cis contacts were defined using MIN_CIS_DIST = 20 kb. Interaction classes were reported, and singletons, multi-mappers, and PCR duplicates were removed (GET_ALL_INTERACTION_CLASSES = 1; RM_SINGLETON = 1; RM_MULTI = 1; RM_DUP = 1). Valid pairs were aggregated into raw contact matrices at 5 kb, 10 kb, 20 kb, 50 kb, 100 kb, 200 kb, 500 kb, and 1 Mb (upper-triangle format). Matrices were then ICE-normalized with up to 100 iterations, filtering low-count bins at 2% and disabling high-count filtering (FILTER_LOW_COUNT_PERC = 0.02; FILTER_HIGH_COUNT_PERC = 0; EPS = 0.1). Default HiC-Pro settings were used otherwise. ValidPairs alignments were converted to the Juicer .hic file format using a combination of HiC-Pro and juicer_tools utilities. Chromatin loops were identified from .hic-formated files using HICCUPs from the Juicer suite [76] of Hi-C tools with non-default parameters (-m512-r1000,2000,5000,1000-f0.5,0.5,0. 5,0.5-p10,8,4,2-i10,8,7,5-d5000,10000,20000,20000--ignore_sparsity).

### scATAC-seq data processing and co-accessibility:

We re-analyzed scATAC-seq data from corresponding leaf tissues for soybean [61], rice [62], and maize [25]. For these datasets, accessible chromatin regions (ACRs) were first identified by creating a pseudo-bulk signal from all cells and calling peaks. A cell-by-peak binary matrix was then constructed, noting the accessibility status of each peak in each individual cell. From this matrix, co-accessibility scores between pairs of ACRs were calculated as previously described [19], using pseudocells generated via a k-nearest neighbors approach [69] to overcome data sparsity. For this analysis, we considered only ACR pairs separated by a minimum and maximum genomic distance of 2kb and 500kb, respectively. The score represents the Spearman correlation coefficient of accessibility between two ACRs across pseudocells adjusted for technical confounders such as read depth. Genic and proximal ACRs were defined as ACRs overlapping (>1bp) gene bodies and within 2kb of TSSs and 500bp of TTS, respectively. All remaining ACRs were classified as distal. Co-expression was determined as pair-wise Pearson correlation coefficient values among all genes expressed (transcripts per million > 1) in at least two independent samples from the maize expression atlas [77].

### Derivation of the polymer physics-based penalty function

#### Data transformation for power-law analysis:

The power-law relationship between contact probability and genomic distance is a basic property of polymer physics. The contact probability *P*(*s*) of two given points on a polymer chain is dependent on the genomic distance s between them. It is also proportional to the probability density Ψ(*R, s*), of finding the end-to-end vector *R* to be zero. This density distribution, in turn, is inversely proportional to the volume *V*(*s*) spanned by the connecting sub-chain in the 3D euclidean space *P*(*s*) ∝ Ψ(*R* = 0, *s*) ∝ *V*(*s*)^−1^. The volume *V*(*s*) scales with the cube of the polymer’s characteristic radius, its root-mean-square end-to-end distance *R_rms_*(*s*), such that *V*(*s*) ∝ *R_rms_*(*s*)^3^. The radius itself scales with the chain length *s* via the Flory exponent *v*, as *R_rms_*(*s*) ∝ *s^v^*. Substituting the expression for *R_rms_*(*s*) into the equation for *V*(*s*), and then into the equation for *P*(*s*), yields the final scaling law, as established in the foundational work of de Gennes [22]: *P*(*s*) ∝ (*s^v^*)^−3^ = *s*^−3*v*^ = *s*^−α^ with *α* := 3*v*.

To characterize the heterogeneous nature of chromatin contact decay, we first processed the Hi-C data for each species. Hi-C loop counts were binned by genomic distance, and both the binned counts and their corresponding distances were transformed into log space (base 10). In this log-log space, a power-law relationship of the form *P*(*s*) ∝ *s*^−*α*^ becomes a linear function with a slope of −*α*.

#### GMM-based decomposition of interaction profiles:

We observed that the log-log distribution of contacts was not perfectly linear, suggesting multiple underlying scaling behaviors. Therefore, a Gaussian Mixture Model (GMM) was applied to the two-dimensional distribution of these log-transformed data points to partition the interactions into distinct subpopulations, each with a unique decay profile (Figure 2A, E, I). The optimal number of components *n* for the GMM was determined for each species by evaluating the Bayesian Information Criterion (BIC) and Akaike Information Criterion (AIC) (Supplementary Information Figure S1). For both Soybean and Rice, the BIC plateaued for models with three to five components; adhering to the principle of parsimony, we selected *n* = 3. For Maize, a single-component model was most appropriate as the BIC reached its local minimum at *n* = 1.

#### Construction of the penalty function:

For each of the *n* clusters identified by the GMM, a linear regression was performed on its constituent log-transformed data points. The power-law exponent *α_i_* for that cluster was calculated from the negative of the resulting slope. This set of decay exponents {*α_i_*} and their corresponding component weights {*π_i_*} from the GMM were used to construct the final penalty function *P*(*s*). This function, which models the expected distance-dependent decay of true chromatin contacts, takes the form of a weighted sum of power-laws:

$$P\left(s\right)=\sum_{i=1}^{n}\pi_{i}\beta_{i}s^{-\alpha_{i}}$$

where Σ *π_i_* = 1 are the weights, *β_i_* are the scaling factors, and *α_i_* the fitted characteristic decay exponents. The specific sets of parameters {*π_i_, β_i_, α_i_*} for each species are provided in Supplementary Table S1.

#### Application to co-accessibility scores:

To generate the final penalized profile, the raw co-accessibility score for each ACR pair was multiplied by the value of the penalty function *P*(*s*) evaluated at the pair’s genomic distance *s*. This procedure selectively down-weights long-range co-accessibility scores that are less likely to represent true physical interactions, thereby aligning the profile more closely with experimental Hi-C data (Figure 1).

### Quantitative metrics for profile comparison

To quantitatively compare the raw and penalized profiles against the experimental Hi-C reference, all profiles were first standardized. They were binned across a consistent genomic distance range of 35 kb to 500 kb. We then employed several metrics to assess performance. To measure the similarity in the shape of the decay curves, we calculated the Spearman’s rank correlation coefficient (*ρ*) to assess the monotonic relationship between a proxy profile and the Hi-C reference. Additionally, we calculated the Wasserstein distance (or Earth Mover’s Distance) to quantify the difference between the two profiles when treated as probability distributions. To quantify the error, we developed area-based metrics. The False Positive Rate (FPR) was defined as the integrated area where a proxy curve’s signal is above the reference curve, normalized by the total area under the proxy curve. Conversely, the False Negative Rate (FNR) was defined as the integrated area where the proxy curve is below the reference, normalized by the total area under the Hi-C reference curve. Finally, to provide a single, consolidated measure of accuracy that balances the trade-off between these two error rates, we calculated the F1 score (Supplementary Table S2).

### Calculation of Deep Learning-based interaction scores

Deep learning (DL)-based interaction scores for maize were generated using GenomicLinks, an open-source tool that predicts chromatin looping potential directly from DNA sequence [19]. The GenomicLinks model utilizes a combination of convolutional neural networks (CNNs) and long short-term memory (LSTM) layers and was previously trained on experimental HiChIP data to learn sequence features indicative of chromatin interaction [48]. We applied the pre-trained GenomicLinks model to all pairwise combinations of previously annotated Hi-C anchor regions across the maize genome. The model produces a raw DL score for each pair, representing the predicted interaction potential. To ensure a direct comparison with our other analyses, these predictions were subsequently filtered, retaining only those pairs separated by a genomic distance within the 0 to 500 kb range. The penalty function was then applied to these filtered scores as described previously.

## Supplementary Material

Supplement 1
