# Supplementary material for: Polymer-derived distance penalties improve chromatin interaction predictions from single-cell data across crop genomes
Source: bioRxiv. 2025 Aug 23:2025.08.20.671329. Preprint. [Version 1] doi: 10.1101/2025.08.20.671329 (PMC12393530; doi:10.1101/2025.08.20.671329)
Supplement: Supplement 1 [file media-1.pdf]

Supplementary Information for:

Polymer-derived distance penalties improve chromatin interaction predictions from single-cell data across crop genomes

Luca Schlegel, Fabio Gómez Cano, Alexandre P. Marand, Frank Johannes

2025

Supplementary Tables

Species	Component (i)	π_i (Weight)	α_i (Decay Exponent)	β_i (Scaling Factor)
**Soybean**	1	0.03	7.16	1.88×10^{35}
	2	0.74	0.31	3.98×10^{3}
	3	0.22	3.91	2.11×10^{13}
**Rice**	1	0.04	6.55	1.80×10^{33}
	2	0.67	0.31	2.42×10^{3}
	3	0.30	3.29	9.17×10^{10}
**Maize**	1	1.00	2.34	5.37×10^{13}

Figure 1: **Supplementary Table S1. GMM-derived parameters for the co-accessibility penalty function.** This table provides the specific parameters used to construct the penalty function $P(s)$. Parameters were derived by fitting a Gaussian Mixture Model (GMM) to the log-log distribution of Hi-C loop counts. The final penalty function is a weighted sum of n power-law components, calculated using the following formula: $P(s) = \sum_{i=1}^{n} \pi_i \beta_i s^{-\alpha_i}$, where for each component i, π_i is the weight, β_i is the scaling factor, and α_i is the decay exponent.

Species	Scenario	FPR (%)	FNR (%)
Soybean	Raw	94.5	3.0
	Penalized	35.1	36.7
Rice	Raw	93.6	3.3
	Penalized	32.4	32.0
Maize	Raw	87.2	0.8
	Penalized	4.5	43.2

Figure 2: **Supplementary Table S2. Area-based error rates for co-accessibility profiles.** This table provides the False Positive Rate (FPR) and False Negative Rate (FNR) for the raw and penalized co-accessibility profiles when compared against the Hi-C ground truth profile. The FPR is calculated as the integrated area where the proxy signal exceeds the Hi-C signal, normalized by the total area under the proxy curve. The FNR is calculated as the integrated area where the proxy signal is below the Hi-C signal, normalized by the total area under the Hi-C curve.

Data	Species	Accession Number(s)	Key Tissue Detail
Hi-C	Maize	GSE120304 / SRP162341	Inner second leaves of 6-day-old seedlings
	Soybean	PRJNA657728	Leaf tissue (wild vs. cultivated)
	Rice	SRP093806	Aerial parts of 10-day-old seedlings
scATAC-seq	Maize	GSE275410	Leaf tissue from ~7-day-old seedlings
	Soybean	GSE270392	Leaf tissue from 10-day-old seedlings
	Rice	PRJNA1007577 / GSE252040	Seedling/leaf tissue

Figure 3: **Supplementary Table S3. Main data sources for Hi-C and scATAC-seq datasets.** Repository accession numbers and key tissue information for all Hi-C and scATAC-seq data re-analyzed in this study.

Supplementary Figures

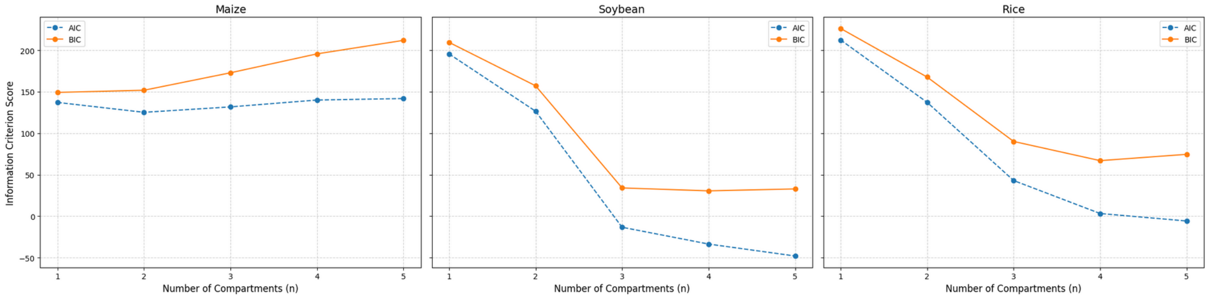

Figure 4: **Determination of the optimal number of components for the Gaussian Mixture Model.** The optimal number of components (n) for the Gaussian Mixture Model (GMM) was determined using the Akaike (AIC, dashed blue line) and Bayesian (BIC, solid orange line) Information Criteria. For Maize, $n = 1$ was selected as it corresponds to the minimum BIC score. For Soybean and Rice, $n = 3$ was chosen as it represents the "elbow" of the BIC curve. In accordance with the principle of parsimony, this point provides the best balance between model fit and complexity to avoid overfitting.

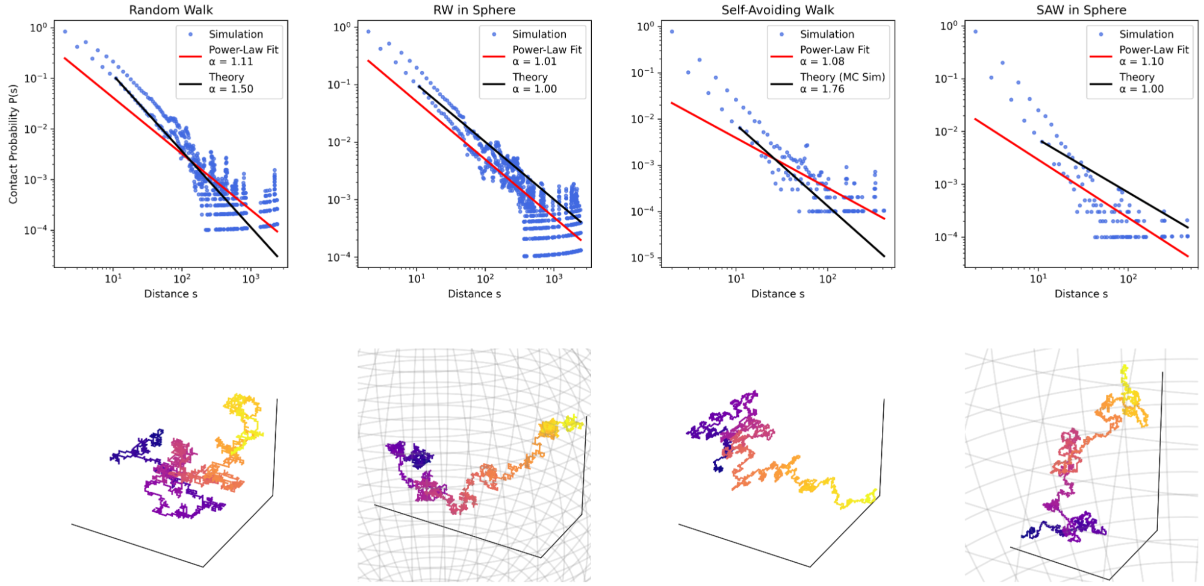

Figure 5: **Comparison of prominent random walks to represent polymer behavior.** Simulations (N=10,000 steps) were performed for a Random Walk (RW) and a Self-Avoiding Walk (SAW), both unconfined and confined within a sphere. The upper panels show the contact probability $P(s)$ as a function of distance s, which follows a power-law $P(s) \sim s^{-\alpha}$. The lower panels show a representative 3D conformation for each model. Discrepancies between these single-run α values and established theory are expected due to stochastic fluctuation. The key takeaway is that a lower α value (closer to 1.0) indicates a more compact state, as seen in the confined walk scenarios. The failure of these simple, uniform models to recapitulate the multi-linear decay observed in experimental Hi-C data underscores the necessity of our multi-component approach.

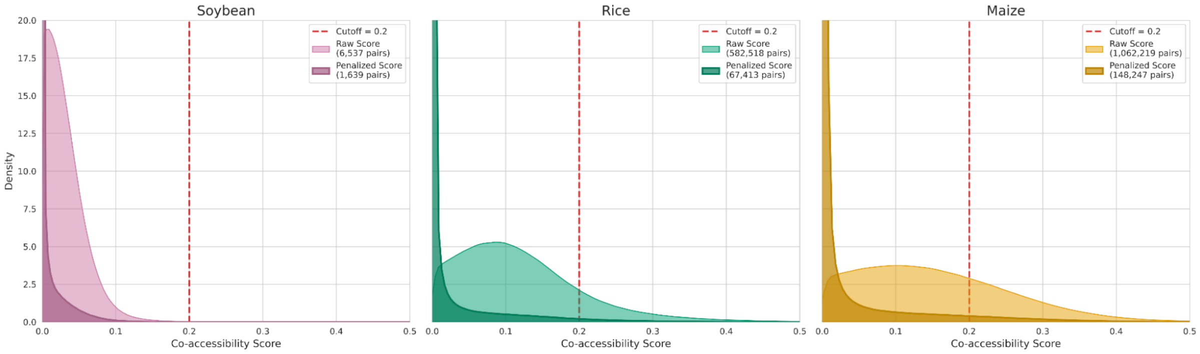

Figure 6: **Co-accessibility distributions are reshaped by a penalty function.** Distributions of raw (light shade) and penalized (dark shade) scores are shown for each species. Applying the penalty reduces the number of pairs with a score greater than an exemplary threshold of 0.2 by approximately 75% in Soybean, 88% in Rice, and 86% in Maize.
